# A web tool for designing and conducting phase I trials using the continual reassessment method

**DOI:** 10.1186/s12885-018-4038-x

**Published:** 2018-02-05

**Authors:** Nolan A. Wages, Gina R. Petroni

**Affiliations:** Division of Translational Research & Applied Statistics, Department of Public Health Sciences, University of Virginia, P.O. Box 800717, Charlottesville, VA USA

**Keywords:** Web application, Dose-finding, Clinical trials, Phase I, Continual reassessment method

## Abstract

**Background:**

Broad implementation of model-based dose-finding methods, such as the continual reassessment method (CRM), has been limited, with traditional or modified 3 + 3 designs remaining in frequent use. Part of the reason is the lack of reliable, easy-to-use, and robust software tools for designing and implementing more efficient designs.

**Results:**

With the aim of augmenting broader implementation of model-guided methods, we have developed a web application for the Bayesian CRM in the R programming language using the Shiny package. The application has two components, simulation and implementation. Within the application, one has the ability to generate simulated operating characteristics for the study design phase, and to sequentially provide the next dose recommendation for each new accrual or cohort based on the current data for the study implementation phase. At the conclusion of the study, it can be used to estimate the maximum tolerated dose (MTD). The web tool requires no programming knowledge, and it is free to access on any device with an internet browser.

**Conclusions:**

The application provides the type of simulation information that aid clinicians and reviewers in understanding operating characteristics for the accuracy and safety of the CRM, which we hope will augment phase I trial design. We believe that the development of this software will facilitate more efficient collaborations within study teams conducting single-agent dose-finding trials.

## Background

Phase I studies are initial safety trials, conducted with the goal of recommending a dose for further testing. Historically, the objective in oncology has been to find the maximum tolerated dose (MTD), defined as the highest dose that can be administered to patients with an acceptable level of toxicity. The toxicity endpoint of interest is usually a binary one, defined in terms of the proportion of patients who experience a dose-limiting toxicity (DLT; yes/no), based on protocol-specific adverse event definitions. In the standard statistical set-up, the MTD is to be chosen from a pre-specified set of dose levels *d*_1_ < *d*_2_ < ⋯ < *d*_*K*_. The majority of design methods are based on the assumption that the probability of a DLT increases with dose, *R*(*d*_1_) < *R*(*d*_2_) < ⋯ < *R*(*d*_*K*_), with the target level of toxicity *θ* typically taking values in the range of 20% to 33%.

The continual reassessment method (CRM [1]) is a model-based method that was introduced as an alternative to the traditional up and down escalation schemes reviewed by Storer [2]. In its original form, the CRM is a Bayesian method that relies on the use of a working dose-toxicity model and a prior distribution to sequentially update the dose-toxicity curve and estimate the dose level at which to treat the next available cohort of patients. It allocates the next patient cohort to the dose level with an estimated DLT rate closest to *θ*. After *n* patients, the MTD is defined as the dose recommended for patient *n* + 1. The CRM assumes a parametric model for the dose-toxicity curve, but it does not require that the model be correct across all the doses under consideration. A one-parameter CRM is under-parameterized and is unlikely to produce a correct fit to the dose-toxicity curve over the entire range of doses. However, as long as the proposed model approximates the relationship reasonably well around the target dose, it will allow for efficient estimation of the MTD. The original CRM paper [1] discussed the use of one- and two-parameter models, but focused primarily on one-parameter models because the simpler models tended to have better properties in terms of identifying the correct MTD.

Statisticians and researchers working on designs for early-phase clinical trials have advocated for increased use of model-based approaches, such as the CRM, to efficiently and accurately address the objectives of finding appropriate doses to merit further research. The operating characteristics of the CRM have been extensively evaluated, and compared to the popular 3 + 3 algorithmic design. Even with evidence that the CRM is the more accurate and efficient design [3, 4], and poses no safety concerns [5], the traditional or modified forms of the 3 + 3 remain the most widely used approaches in dose-finding studies [6–8]. A monograph on the CRM [9] attributes the infrequent implementation of CRM to several real or perceived difficulties. The first is a perception that the method is computationally burdensome, leading to clinician and reviewer uncertainty about how the design works and creating a mentality that the method is a “black box” of allocation decisions. Not unrelated to the notion of complexity, is the impression that the CRM is quite sensitive to the choice of design specifications, such as a working dose-toxicity model and a prior distribution, which further contributes to its infrequent use. However, recent work in the area to address these impressions [9–15] has been made in an attempt to overcome this hurdle by offering practical recommendations that can be applied in a broad range of situations. These recommended specifications yield desirable operating characteristics for a wide variety of commonly encountered practical settings.

With the general reluctance to produce protocol specific CRM programs, use of the CRM is limited by a lack of accessible software tools for designing and conducting these trials. This reluctance can be overcome by continued development of accessible software using the simple and practical recommendations while minimizing the design specification choices. Several statistical software packages have been developed for the CRM, but these programs require a certain level of programming knowledge to operate. With the aim of providing a user-friendly web interface for designing and conducting phase I trials using the Bayesian CRM, we have developed an R Shiny web application that relies on the practical design specifications for the statistical components of the method. We hope the availability of this software will facilitate the use of the Bayesian CRM in future dose-finding studies.

## Implementation

The application relies upon the following set of default statistical specifications.

### Default statistical parameters

#### Choice of working model

The most common model choice implementation in the CRM uses the “empiric” model to model the DLT probabilities *R*(*d*_*k*_) at each dose. This parametrization raises a set of initial DLT probability estimates, also referred to as the “skeleton” of the model, to a power exp(*a*) so that

$$ R\left({d}_k\right)=\Pr \left(\mathrm{DLT}\ \mathrm{at}\ \mathrm{dose}\ {d}_k\ \right)\approx {\alpha}_k^{\exp (a)}, $$

where *α*_*k*_ are pre-specified constants (skeleton) of the model and *a* is the model parameter to be estimated by the data. Paoletti and Kramar [10] provide a comprehensive comparison of various working model choices in the CRM. These comparisons support that a one-parameter model should be used and that the use of the empiric model is sufficient and provides satisfactory performance in the vast majority of situations. The authors [10] generated 5000 dose-toxicity curves and found that the one parameter empiric model had superior properties to the two-parameter logistic model. For further discussion on the impact of over-parameterization in the CRM, we refer the reader to Iasonos et al. [11].

#### Skeleton choice

The skeleton does not have to be related to the actual doses or the probabilities of DLTs at the actual doses, but rather is selected to yield good operating characteristics of the CRM as described in Lee and Cheung [12]. It has been shown by several authors [12, 13] that CRM designs are robust and efficient with the implementation of “reasonable” skeletons. O’Quigley and Zohar [13] define a “reasonable” skeleton as one that demonstrates good robustness properties in terms of its operating characteristics. It is relatively straightforward to have an intuitive idea about whether or not a skeleton is “reasonable.” For instance, the “unreasonable” skeleton *α*_*k*_ = {0.12, 0.20, 0.21, 0.22, 0.36} would have trouble distinguishing between levels 2, 3 and 4. Similarly, the skeleton *α*_*k*_ = {0.01, 0.20, 0.85, 0.90, 0.95} would encourage experimentation at level 2 when targeting a DLT probability of *θ* = 0.20. This will likely have poor operating characteristics if the true MTD is any dose other than level 2.

To generate reasonable skeletons, we can rely on the algorithm of Lee and Cheung [12] to produce adequate spacing between skeleton values at neighboring doses, without having to rely on a clinician’s estimate at every dose level. The algorithm is available as a function, **getprior**, within the R package **dfcrm** and requires four pieces of information in order to generate the skeleton; the prior MTD (*ν*), the target toxicity rate (*θ*), the number of dose levels (*K*), and a spacing measure (*δ*) of the skeleton. The values of *θ* and *K* are pre-specified. Pan and Yuan [14] showed that skeletons produced by the algorithm of Lee and Cheung [12] are invariant to the specification of the prior location of the MTD *ν*, indicating that skeletons obtained by using different values of *ν* are equivalent. The work conducted in calibrating a skeleton and prior in Bayesian CRM [12, 15] has been focused on setting the prior MTD to be the median dose, so we lean on this approach in the web application. As for *δ*, based on simulation results in Cheung [9], the optimal range of *δ* is [0.04, 0.08] for *θ* = 0.20, 0.25 and [0.04, 0.10] for *θ* = 0.33. A value of *δ* = 0.05 lies in the optimal range for common values of *θ*, and thus will result in reasonable skeletons in many practical situations. Using this information, we can generate skeleton values using the **getprior** function in R package **dfcrm** (i.e *getprior*(*δ*, *θ*, *ν*, *k*)).

#### Prior distribution on the model parameter

For the empiric model, O’Quigley and Shen [16] recommend the use of a mean zero normal prior $N\Big(0,{\sigma}_a^2$). The standard deviation *σ*_*a*_ completely specifies the prior distribution, and Lee and Cheung [15], as well as Chapter 9 in Cheung [9], describe a technique for calculating the least informative normal prior for use in the Bayesian CRM. This prior distribution is vague in terms of which dose is the MTD [15], and we incorporate this calibration method into the app.

#### Other design specifications

Two other important design specifications need to be noted for inclusion into the statistical section of a protocol document.

1. **Dose escalation skipping restriction**: The trial is not allowed to skip dose levels when escalating.
2. **Stopping rule for safety**: The trial stops for safety if the lowest dose is indicated by the data to be too toxic. Specifically, with the procedure used in the application, the trial stops for safety if the lower limit of a 90% probability interval [17] exceeds the target DLT rate.

## Results

The web application is written in the R programming language [18] and is made freely available using the Shiny package [19]. Access to the application online is available at https://uvatrapps.shinyapps.io/crmb/. The R code for the application can be downloaded by locating the ‘R code’ section at http://faculty.virginia.edu/model-based_dose-finding/. The application has a simple web interface with the capability to:

1. Simulate operating characteristics for the Bayesian form of the CRM,
2. Compute the recommended dose level for the next patient cohort based on accumulated data,
3. At the conclusion of the study, it can be used to estimate the maximum tolerated dose (MTD).

The simulation function generates the operating characteristics of the Bayesian CRM based upon the user specifying the following set of input parameters (Table 1).

1. The true DLT probability at each dose level.
2. The target DLT probability that defines the MTD for the study.
3. The number of patients to be accrued to the study before the next model-based update. Note, cohort size may be 1, 2 or 3 patients.
4. Enter the maximum sample size for the study. This number should be a multiple of the cohort size entered in the previous input line
5. Enter the total number of patients treated on any dose required to stop the trial. At any point in the trial, if the recommendation is to assign the next cohort to a dose that already has the entered number of patients treated on the dose, the study is stopped and the recommended dose is declared the MTD. If the entered number is larger than the maximum sample size, each trial will accrue to the maximum sample size.
6. Enter the number of simulations. A minimum of 1000 is recommended [20, 21]
7. Enter the index of the starting dose level. Note: The index of lowest dose level is always 1. If the design allows for ‘minus’ dose levels (i.e. -2, − 1 dose levels), then the index of the starting dose should account for these lower levels (i.e. if a − 1 dose level is allowed, the index of the starting dose is 2.)
8. Set the seed of the random number generator.

**Table 1:**
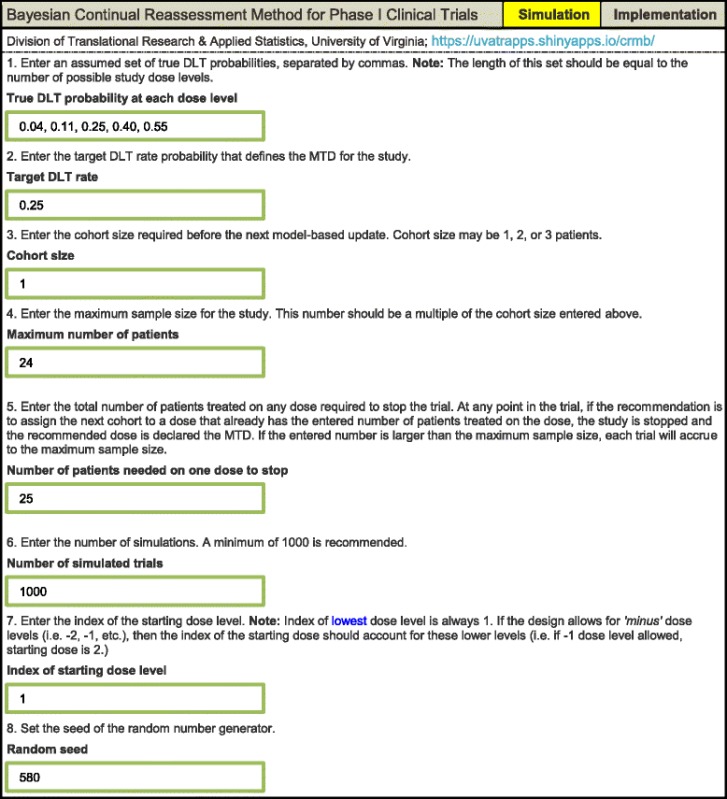
User input for the simulation component of the continual reassessment method (CRM) web application

The simulation results will be generated by clicking the *Run Simulation Study* button. It is also of interest for investigators to be able to conduct a trial with the Bayesian CRM using the app. That is, given accumulated DLT data for all patients on each dose level, what dose would be recommended for the next entered patient cohort, targeting a *θ* DLT rate? For implementation, the app relies upon the user specifying the following set of input parameters (Table 2):

**Table 2:**
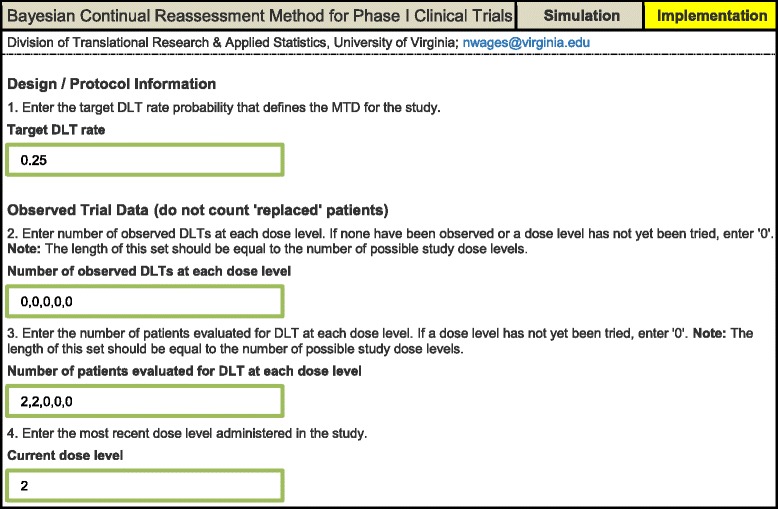
User input for the implementation component of the continual reassessment method (CRM) web application

*Design / protocol information*

1. The target DLT probability that defines the MTD for the study.

*Observed trial data (do not count ‘replaced’ patients)*

2. Enter the number of observed DLTs at each dose level. If none have been observed or a dose level has not yet been tried, enter “0.” **Note**: The length of this set should be equal to the number of possible study dose levels.
3. Enter the number of patients evaluated for DLT at each dose level. If a dose level has not yet been tried, enter “0.” **Note**: The length of this set should be equal to the number of possible study dose levels.
4. Enter the most recent dose level administered in the study.

The implementation results will be generated by clicking the *Get Next Recommended Dose* button.

### Simulation results

Of particular interest in simulating operating characteristics is the accuracy of the method under an assumed set of true DLT probabilities and target DLT rate. Accuracy is typically measured by the percentage of simulated trials in which the true MTD is recommended as the MTD at the conclusion of the study. This is commonly termed the percentage of correct selection (PCS). Also of interest is the safety of the design, which is typically evaluated by how patients are allocated. Safety can be assessed through observing how many patients were allocated, on average, to dose levels at and around the true MTD, as well as by how many patients, on average were treated above the true MTD. Based on the simulation input provided by the user, the application will produce operating characteristics for the Bayesian CRM using the default statistical parameters described in Methods section. The results output:

1. The skeleton of the working model used,
2. the true DLT probability at each dose level,
3. the percentage of trials in which each dose was selected as the MTD,
4. the average number of DLTs observed at each dose level,
5. the average number of patients treated at each dose level,
6. the percentage of trials stopped for safety, based on the safety stopping rule described in the Methods section.

As an example, consider the input specifications in Table 1. Based on 1000 simulated trials of 24 patients, the output in Table 3 is generated. These tables can be copied and pasted into a protocol document. The skeleton used in each simulated trial is {0.08, 0.16, 0.25, 0.35, 0.46}. Targeting *θ* = 0.25, the assumed MTD under the set of true DLT probabilities is dose level 3, with true DLT probability of 0.25. Dose level 3 is selected as the MTD in 60.2% simulated trials, while 11.3 of 24 patients on average are treated at the true MTD in this scenario. CRM simulations using R packages **dfcrm** and **bcrm** yield very similar results. Using the same specifications, dose level 3 is selected in 60.2% and 60.3% of simulated trials by **dfcrm** and **bcrm**, respectively. On average, 11.29 and 11.30 of 24 patients are treated at the true MTD for the **dfcrm** and **bcrm**, respectively. Using our app, an average of 2.8 DLTs are observed at the true MTD. The results in Table 3 can be reproduced exactly by any user by inputting the exact same design specifications, provided that the same random seed is used to generate the outcomes in each simulated trial.

**Table 3:**
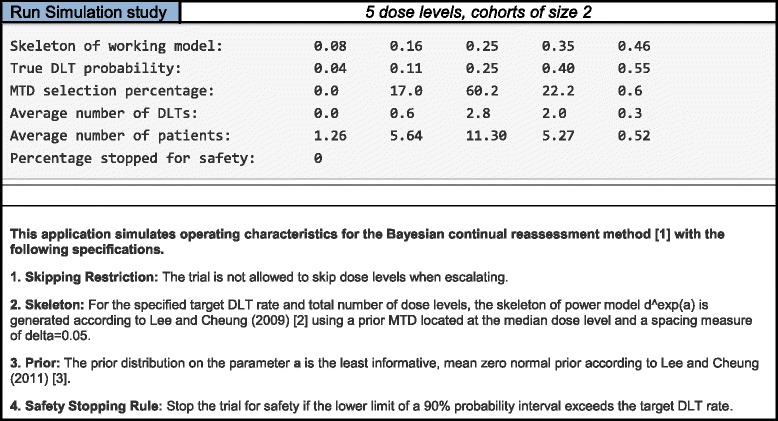
Output for the simulation component of the continual reassessment method (CRM) web application

### Implementation results

Tables 4 illustrates the application’s ability to update model-based estimates for the DLT probabilities and the recommended dose for the next entered cohort. Suppose we begin a study at dose level 1, and are accruing to the study in patient cohorts of size 2. The target DLT rate is *θ* = 0.25, and the first two entered patient do not experience a DLT (Table 4). Based on the two non-DLT observations at dose level 1, we can see that the estimated DLT probabilities have been updated, indicating that dose level 3 is closest to the target dose, with estimated DLT rate 0.21. However, the method implements a dose escalation restriction on skipping dose levels when escalating, so the *Recommended dose level* in Table 4 is level 2.

**Table 4:**
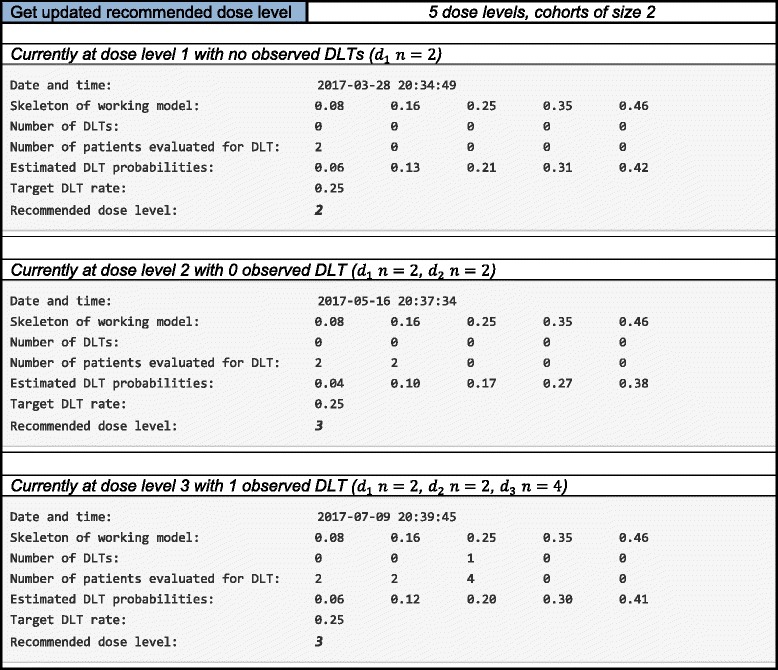
Output for the implementation component of the continual reassessment method (CRM) web application. After the accrual of each new patient cohort, the model-based DLT probability estimates and recommended dose level for the next accrued cohort are updated

The user would then update *Current dose level* in the input parameters to dose level 2, and observe the DLT outcome (yes/no) for the second entered patient cohort. Suppose these patients also did not experience DLTs. Based on these additional non-DLT observations at dose level 2, we can again see that the estimated DLT probabilities have been updated, indicating that dose level 4 is closest to the target dose, with an estimated DLT rate of 0.27. The method’s dose escalation restriction is again triggered, making the *Recommended dose level* in Table 4 dose level 3. The final entry in Table 4 illustrates the model-based recommendation being dose level 3, after 1/4 DLTs have been observed at dose level 3. Given the same data, the results in Table 4 can be verified using the **dfcrm** package in R, thus validating the code used for the app. The implementation portion of the application can be used to sequentially provide model-based dose recommendations for trial conduct in real studies. It is also useful in providing tables of the early design behavior in the protocol statistical section, so that reviewers get an idea of how the design allocates early in the study. The date and time each implementation output was generated is given at the top of the output, so that each recommendation can be properly documented.

## Conclusions

In this article, we have presented software in the form of an R Shiny web application for simulating and conducting Phase I trials using the Bayesian form of the CRM. The web tool provides a mechanism for conducting the Bayesian CRM in a timely and reproducible fashion, requiring no programming knowledge. It utilizes a set of default design specifications based on practical recommendations from literature. These specifications produce robust operating characteristics. The app contains the type of simulation information that aid clinicians and reviewers in understanding operating characteristics for the accuracy and safety of the CRM. A quick comparison can be made to the operating characteristics of the 3 + 3 using the R Shiny web application of Wheeler, Sweeting, and Mander [22], as well as to a non-parametric optimal benchmark [23]. The bottom of our web page contains detailed notes about the design specifications, including the skipping restriction and safety stopping rule, which can be input into a protocol statistical section. The app is free to access and use on any device with an internet browser, including a smart phone. We hope this leads to broader implementation of model-based designs and will facilitate more efficient collaborations within study teams.

## Availability and requirements

Project name: CRM web application.

Project home page: https://uvatrapps.shinyapps.io/crmb/

Operating system(s): Platform independent.

Programming language: R.

Other requirements: version 2.8.1 or later.

License: GPL-2.

Any restrictions to use by non-academics: none

## Abbreviations

CRM: continual reassessment method
DLT: dose limiting toxicity
MTD: maximum tolerated dose
PCS: percentage of correct selection
